# KRAS Mutations in Cancer: Understanding Signaling Pathways to Immune Regulation and the Potential of Immunotherapy

**DOI:** 10.3390/cancers17050785

**Published:** 2025-02-25

**Authors:** Priyanka Uniyal, Vivek Kumar Kashyap, Tapan Behl, Deepak Parashar, Ravi Rawat

**Affiliations:** 1 Department of Pharmaceutical Technology, School of Health Sciences and Technology, UPES, Dehradun 248007, India; priyanka.113628@stu.upes.ac.in; 2 Division of Cancer Immunology and Microbiology, Medicine, and Oncology Integrated Service Unit, School of Medicine, University of Texas Rio Grande Valley, McAllen, TX 78504, USA; vivek.kashyap@utrgv.edu; 3 South Texas Center of Excellence in Cancer Research (ST-CECR), School of Medicine, University of Texas Rio Grande Valley, McAllen, TX 78504, USA; 4 Amity School of Pharmaceutical Sciences, Amity University, Mohali 140306, India; tapanbehl31@gmail.com; 5 Division of Hematology & Oncology, Department of Medicine, Medical College of Wisconsin, Milwaukee, WI 53226, USA

**Keywords:** cancer, KRAS mutation, immunotherapy, oncogene, tumor microenvironment

## Abstract

The Kirsten rat sarcoma viral oncogene homologue (KRAS) mutation is one of the most prevailing mutations in various tumors and is difficult to cure. Long-term proliferation in carcinogenesis is primarily initiated by oncogenic KRAS-downstream signaling. Recent research suggests that it also activates the autocrine effect and interplays the tumor microenvironment (TME). Here, we discuss the emerging research, including KRAS mutations to immune evasion in TME, which induce immunological modulation that promotes tumor development. This review gives an overview of the existing knowledge of the underlying connection between KRAS mutations and tumor immune modulation. It also addresses the mechanisms to reduce the effect of oncogenes on the immune system and recent advances in clinical trials for immunotherapy in KRAS-mutated cancers.

## Simple Summary

KRAS mutations promote oncogenic signaling and modify the tumor microenvironment (TME), promoting immune evasion and tumor growth. These alterations lead to treatment resistance, highlighting the importance of targeted therapies in KRAS-driven cancers. This review explores the complex function of KRAS in cancer development, highlighting its influence on immune regulation and resistance to therapy. We address novel ways to mitigate these effects, notably immunotherapies and targeted medicines to suppress KRAS-driven signaling. Emerging clinical innovations highlight the possibility of integrating immune-based treatments with KRAS inhibitors to improve therapeutic success. Addressing KRAS-induced immune alteration may facilitate precision medicine strategies, enhancing patient outcomes in KRAS-mutant cancers.

## 1. Introduction

Cancer is a pathological condition characterized by the uncontrolled division and proliferation of cells, often resulting from genetic mutations in certain genes [1,2]. Uncontrolled proliferation, adhesion, and metastasis are the major identifying characteristics of these cells. It is the second largest cause of mortality worldwide, contributing to approximately 10 million deaths, or one out of six deaths, in 2020. These genetic alterations in oncogenic signaling promote cancer development by stimulating proliferation while decreasing apoptosis in cancer cells. During the last few years, the emerging literature indicated that some oncogenic mutations regulate immune system interactions through oncogenic signaling [3]. Several oncogenes that are functionally active in malignancies have been reported. The RAS (rat sarcoma viral) oncogene is the earliest and most broadly examined among them [4]. RAS mutation contributes to different human cancers being a genetic molecular mediator and is found to be involved with fundamental mechanisms such as uncontrolled cell proliferation, evasion of apoptosis, metabolic reprogramming, and immune evasion. It induces several signaling-pathway stimulations, including Ral guanine nucleotide exchange factors (RAL-GEF), mitogen-activated protein kinase (MAPK), and phosphoinositide 3-kinase (PI3K). These pathways stimulate critical cellular activities, including cell differentiation, proliferation, and survival of extracellular stimuli [4,5]. The most common and frequently mutated RAS oncogene family comprises the exceptionally identical genes neuroblastoma RAS (NRAS), Harvey RAS (HRAS), KRAS4A, and KRAS4B, which are observed as activated mutated genes in over 25% of cancers (version 91, COSMIC database). The isoform of KRAS is the target of most mutations (86%), where distribution and prevalence differ based on different types of cancer. The widely recognized oncogene, KRAS, has the maximum mutation rate and correlates with various kinds of cancers that are extremely fatal, such as pancreatic ductal adenocarcinoma (PDAC, 98%), colorectal cancer (CRC, 52%) and lung adenocarcinoma (LAC, 32%) [6]. The establishment of targeted inhibitors and tumor-driver genes has completely transformed cancer therapy techniques and clinical results [7,8,9].

A complex interrelationship between cancer cells and infiltrating immune cells is regulated by molecular processes in the tumor microenvironment (TME) [6,10]. Immune cells can function in a pro-tumorigenic manner, in addition to being involved in the identification and removal of cancer cells via the immune system [10,11]. The TME consists of stromal cells, including endothelial cells, fibroblasts, adipocytes, and the extracellular matrix (ECM), along with innate immune cells such as natural killer (NK) cells, macrophages, neutrophils, myeloid-derived suppressor cells (MDSCs), CD8^+^ T cells, CD4^+^ T cells, and dendritic cells [12]. Various types of cells inside the complicated and diverse environment may modulate cancer development by the production of chemokine and cytokine in an autocrine and paracrine mode [10]. RAS oncogene mutations have been predicted to have a transforming role due to their self-efficiency in the growth signals. Therefore, an emergence in awareness of carcinogenicity and its fundamental mechanisms demonstrates the impacts of oncogenic RAS mutation expand above its potential for persistent multiplication. It is now wholly understood that KRAS oncogenic mutations facilitate autocrine signaling and interactions with the TME, specifically by immune response evasion and eventually resulting in cancer development, invasion, and progression [13,14]. The KRAS oncogene generated in cancerous cells modifies the adjacent stromal cells to produce these effects via activating several chemicals, namely chemokines, cytokines, and growth factors. Furthermore, to produce an immunosuppressive stroma, oncogenic KRAS interacts with mutations in the oncogene or tumor-suppressor gene [15].

This review addresses the most recent advancements in the aspects of KRAS, the attributes of KRAS mutations in cancer patients, and its correlation with tumor immunity and emphasizes the immunomodulatory pathways in cancer and immunotherapeutic approaches to target KRAS-related mutations in different cancers.

## 2. Kirsten Rat Sarcoma Viral Oncogene Homologue (KRAS) and Signal Transmission

### 2.1. Structure of KRAS

The KRAS gene belongs to the RAS oncogene family, which possesses two distinct human isoforms: neuroblastoma and Harvey rat sarcoma viral oncogene (NRAS and HRAS). Barbacid and Weinberg discovered a gene from human bladder cancer in carcinoma cells in 1982. The HRAS gene, identified from the short arm of chromosome 11 (11p15.1–11p15.3), has been subsequently demonstrated to be the human homolog of RAS genes [16]. A different homolog was identified from the short arm of chromosome 12 (12p11.1–12p12.1), known as KRAS, and was discovered in human lung cancer cells. The last gene, NRAS, is localized on the short arm of chromosome 1 (1p22–1p32) and is identified in human neuroblastoma [17,18,19]. RAS genes are comprised of four exons uniformly grouped throughout the entire length of around 30 kb of DNA and have similar structures that have remained preserved throughout evolution. Due to the different clippings of the fourth exon, KRAS genes encoded two relatively similar protein isoforms, namely KRAS 4A and 4B, which comprise 189 and 188 amino acids, respectively [20]. Due to the excess of mRNA that encodes KRAS-4B in the cell, the term KRAS is usually called KRAS-4B [21]. The remaining two RAS proteins consist of 189 amino acids.

RAS is a variety of membrane-bounded regulatory proteins (G protein) primarily attached to guanine nucleotides, which is associated with the guanosine triphosphatase family (GTPases) [22]. The function of RAS is to act as a binary switch between guanosine diphosphate (GDP) and triphosphate (GTP), which mediates essential signal transmission through receptors on the membrane to the intracellular molecule [23]. Because of the intrinsic GTPase activity, it further hydrolyzes the GTP to GDP, and KRAS commonly attaches to the GDP in its inactive form [24]. The KRAS-GDP complex shows less affinity for GDP in the presence of guanine nucleotide exchange factors (GEFs) after cellular stimulation and the interactions between epidermal growth factor (EGF) and its receptor (EGFR). This process promotes the exchange of GDP to GTP within the protein, utilizing higher intracellular concentrations of GTP (about 10-fold) and its greater binding affinity relative to GDP [25].

**Figure 1:**
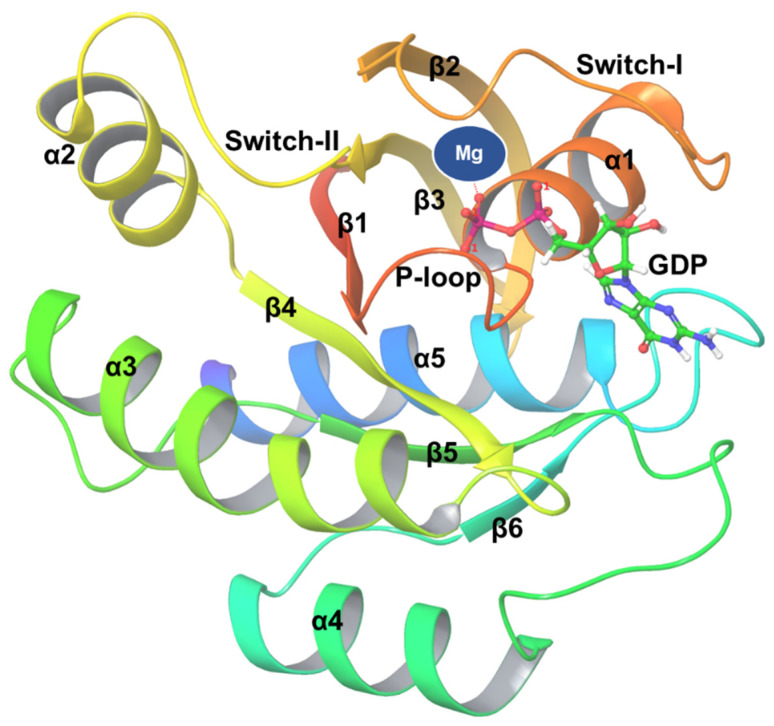
Crystal structure of human KRAS protein bound with GDP (PDB ID: 4OBE).

The G domain of KRAS, consisting of residues 1–166, is essential in regulating the physiological function of GTPase proteins [26]. Five alpha helices and six beta strands are observed in the crystal structure of the KRAS [27] Protein Data Bank Identification Code (PDB ID: 4OBE), and together, they formed the G domain, referred to as the catalytic domain. The other domain formed is a hypervariable area (HVR), which consists of membrane localization-related CAAX motif [20,21] (Figure 1). Switch I, switch II, and P-loop, are the three regions that constitute the G domain of KRAS, which primarily link to a guanine nucleotide with signaling stimulation via binding with effectors and RAS regulators [28,29]. Guanine nucleotide exchange factors (GEFs), like son of sevenless (SOS) and GTPase-activating proteins (GAPs) like Neurofibromin 1 (NF1), are two distinct parts of regulatory proteins that are essential for analyzing these binary switches [30]. Switches I and II of the G domain completely alter the configuration of KRAS-GTP binding, as they stimulate KRAS and bind toward its downstream molecules like a monomer or dimer to regulate the multitude of signaling transduction pathways. On the other hand, GAPs maintain the inactive state of KRAS by elevating its GTPase activity, hence boosting the binding among both GDP and KRAS [31].

**Figure 2:**
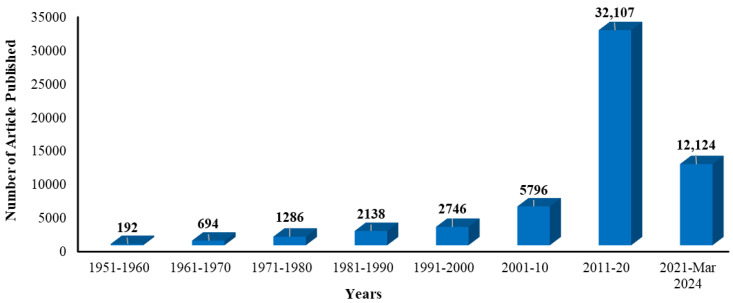
Number of articles published on KRAS protein associated with different types of cancer.

It is important to note that many definitions of residues are applied to switch domains. Such definitions are somewhat imprecise because the switch domains have a significant related flexibility. In switch II phrases, the starting point is located among residues 58–60, and the endpoint is located between residues 67 and 76. The helix α2 is either entirely or partially included or excluded. For illustration, residues 30–40 are defined as switch I, residues 58–72 are defined as switch II, and residues 10–14 are defined as P-loop. It is to be noted that the P-loop, also referred to as the Walker A motif [32], extends up to S17 [33]. Regions of frequent mutation in cancer have been identified in either the switch II region or in the P-loop. Several KRAS protein structures have been deposited in the Protein Data Bank (Table 1). The first structure was unveiled in 1996, and since then, the count of KRAS structures has consistently been generated, with dozens being included every year. Nearly fifty percent of these structures are attached to GDP, while around one-third are attached to GTP or its non-hydrolyzable analogs like GCP, GNP, or GSP. Certain structures do not properly bind nucleotides, such as those that consist of HVR peptides, competitive ligands for GDP/GTP, or nucleotide exchange groups like KRAS-SOS1. These structures also contain a diverse range of mutations. The prevalent oncogenic KRAS mutations include the G12A, G12C, G12D, G12R, G12V, G13D, and Q61H structures [34]. Because of its critical involvement in cancer biology, KRAS is frequently referred to as the Holy Grail of therapeutic discovery [35] and has been claimed to be a key oncogenic driver for drug development [36] (Figure 2). Table 1 lists some inhibitors, such as AMG 510 (sotorasib), which are approved by the US FDA as KRAS G12C inhibitors.

### 2.2. KRAS Upstream Regulation

KRAS upstream signaling pathways are initiated by the interaction of ligands to its transmembrane receptors, generally receptor tyrosine kinases, such as GRB2, in association with RAS-specific GEFs, which enhances KRAS activation [37,38,39]. KRAS bound with GTP significantly transmits the signal into its downstream pathway and thereby stimulates several signaling pathways [40,41,42,43,44,45,46,47]. Therefore, KRAS is found to regulate several cellular functions, including cell survival, development, cell division, proliferation, and apoptosis [48,49,50].

#### 2.2.1. GRB2-SOS1 Complex

Receptor tyrosine kinase (RTKs) can be triggered via different growth factors, namely epidermal growth factors (EGF), fibroblast growth factors (FGFs), and platelet-derived growth factors (PDGF). Sequentially, RTKs activate the KRAS protein using an intermediary molecule. An instance of such occurs when EGF communicates with EGFR, leading to the phosphorylation and dimerization of EGFR [48]. An intermediary molecule, growth factor receptor-bound protein 2 (GRB2), binds phosphorylated EGFR by employing its own SH2 domains. Two SH3 domains and one SH2 domain constitute GRB2, and the SH3 domain potentially binds SOS1, a type of GEF. The linkage between KRAS and GTP is facilitated by activated SOS1 which further modifies the KRAS from its inactive form to its active form. As previously indicated, KRAS forms are mainly aligned with GEFs/GAPs. Moreover, the KRAS upstream molecule primarily mediates its activation or inactivation via monitoring these two molecules. Tulpule and his team recently introduced a new, membrane-independent methodology to stimulate KRAS [49]. Certain fusion proteins comprising RTKs, including EML4-ALK, may bind with GRB2 and SOS to consistently activate KRAS and stimulate downstream signals to develop membrane-less cytoplasmic granular proteins. Remarkably, this strategy is closely related to, but not similar to, phase separation.

#### 2.2.2. RAS-GRF1

Another GEF, identified as RAS protein-specific guanine nucleotide-releasing factor (RAS-GRF1), is mainly produced in the brain. When glutamate, including N-methyl-D-aspartic acid receptor (NMDAR), transmits signals to the KRAS in mature neurons, this stimulates the subsequent MAPK/ERK cascade response [50]. The RAS-GRF1’s capacity to stimulate KRAS was substantially increased with emerging Ca^2+^ concentrations, demonstrating mutual regulations and innermost connections among KRAS signaling and Ca^2+^ signaling together with the metabolism [51,52]. Additionally, when the chemokine receptor binds in the cell membrane, it may activate the protein kinase A by introducing cAMP. RAS-GRF1 can be activated through phosphorylation by activated protein kinase A, thus elevating the KRAS transformation from an inactivated form to an activated form [53].

#### 2.2.3. SHP2

As previously specified, GEFs are an important molecule for the activation of KRAS. Src homology phosphatase 2 (SHP2) shows a crucial function in KRAS activation [54,55]. PTPN11 encodes SHP2, which is protein tyrosine phosphatase (PTP), and contains Src homology 2 domains (SH2) [56]. Unlike other PTPs, several findings suggested that SHP2 has a significant function in the activation of the pathway related to intracellular signaling, primarily the KRAS/ERK pathways [55]. The SHP2 is a commonly used signaling receptor that delivers a series of tyrosine kinase receptor-responsive signalings to the KRAS-ERK signaling pathway [57]. Additionally, SHP2 may facilitate the attachment of GRB2-SOS1 to the receptor. Several findings suggested that specific SHP2 phosphorylation sites serve as the prime binding sites for GRB2, like the 580 and 542 of SHP2 [58]. As an outcome, SHP2 has a scaffolding function on the recruitment of GRB2. On the other hand, the enzymatic activity of SHP2 was necessary to enhance the KRAS activation by the substrate SHP2’s dephosphorylation [59]. Several substrates of SHP2 dephosphorylation have been demonstrated as improvers of the activation of KRAS. This activation function is also mediated adversely by a few molecules, including the GTPase-activated protein p120-RASGAP. It was previously found that SHP2 targets RASGAP and minimizes the negative regulatory impact of p120-RASGAP39 by the RasGAP-p120 junction dephosphorylation with modulating p120-RASGAP engagement near KRAS [60,61]. SHP2, with p120-RASGAP, serves as a signaling molecule through multiple molecules’ dephosphorylation, including sprouty activators and negative regulators, by the Src regulatory proteins’ dephosphorylation [62,63].

### 2.3. Mutation Patterns of KRAS

KRAS mutations show tumor type-specific patterns, with G12D and G12V being the most frequent among cancers, but they vary in frequency. In particular, G12D is prevalent in pancreatic cancer, while G12V is prevalent in ovarian cancer. G12C, remarkable for its predominance in lung adenocarcinoma (~40%), is significantly less frequent in other cancers (~10%) [64,65]. Other mutations, such as G12A in endometrial cancer, G12S in cholangiocellular cancer, and G12R in pancreatic cancer, reveal unique patterns connected to carcinogenic mechanisms such as DNA repair deficits. Smoking-associated mutations (e.g., G12C) and mismatch repair deficits (e.g., G12D, G13D) further illustrate the etiological diversity of KRAS mutations [66,67]. Driver analyses reveal G12C as a prominent driver in lung cancer, G12R in pancreatic cancer, and G12V/G12D in endometrial cancer, underlining the therapeutic and preventative significance of mutation-specific roles in carcinogenesis [68,69].

### 2.4. KRAS-Mediated Signaling Pathway

#### 2.4.1. The RAF-MEK-ERK Pathway

The authoritative downstream-targeted KRAS signaling is the RAF-MEK-ERK pathway [70]. Threonine/serine-specific protein kinase characterized as rapidly accelerating fibrosarcoma (RAF), which can be activated by heterologous or homologous dimerization, can be recruited by activated KRASGTP on the plasma membrane from the cytoplasm, and leads to the configurational alterations in RAF [71]. The stimulation of MEK1/2 occurs via the binding of the C-terminal catalytic domain of RAF through phosphorylation, which further activates ERK1/2. The activated ERK phosphorylates the E26 transformation-specific transcription factors (ETS), the serum response factor (SRF), the ETS like-1 protein, and the ribosomal S6 kinase (RSK) to mediate the translation and transcription of the respective targeted genes. Such processes contribute to regulating cell mobility, differentiation, and proliferation, and several life processes [71,72,73]. 

#### 2.4.2. The PI3K-AKT-mTOR Pathway

KRAS was recognized as being engaged in the PI3K-AKT-mTOR pathway, which is essential for cell life functions, including cell division, apoptosis, proliferation, and glucose transportation, and shows a significant impact on the progression of tumor resistance [41]. Activated KRAS binds to the p110 subunit of PI3K for its activation [74]. Furthermore, activated PI3K was transformed and catalyzed to phosphatidylinositol 3,4,5-trisphosphate (PIP3) by phosphatidylinositol 4,5-bisphosphate (PIP2) [75]. PIP3 is stimulated to phosphorylate AKT at Thr308 by phosphoinositide-dependent kinase 1 (PDK1), whereas mTOR complex 2 eventually phosphorylates AKT at its serine phosphorylation site (Ser473) [76]. Phosphatidylinositol 3,4,5-trisphosphate (PIP3) develops when phosphatidylinositol 4,5-bisphosphate (PIP2) are catalyzed by activated PI3K. AKT is phosphorylated at Thr308 via phosphoinositide-dependent kinase 1 (PDK1) with the support of PIP3. AKT activates completely when the mTOR complex 2 phosphorylates the serine phosphorylation site (Ser473). Activated AKT reaches the nucleus, stimulates or inhibits various downstream pathways, and modulates metabolic and cellular growth functions [77]. Furthermore, AKT can effectively stimulate the mTOR target protein, which plays a significant role in cell survival, metabolism, proliferation, transcription, and protein synthesis [78]. Additionally, Bcl-XL/Bcl-2-associated death promoters (BADs) are activated and phosphorylated through AKT, resulting in BAD binding with the companion protein 14-3-3 rather than Bcl-2/Bcl-XL and, hence, inhibiting the apoptosis [79].

## 3. KRAS-Mutated Genes and Their Impact on Cancer Progression

### 3.1. Diverse KRAS Co-Mutations in Tumorigenesis

**Figure 3:**
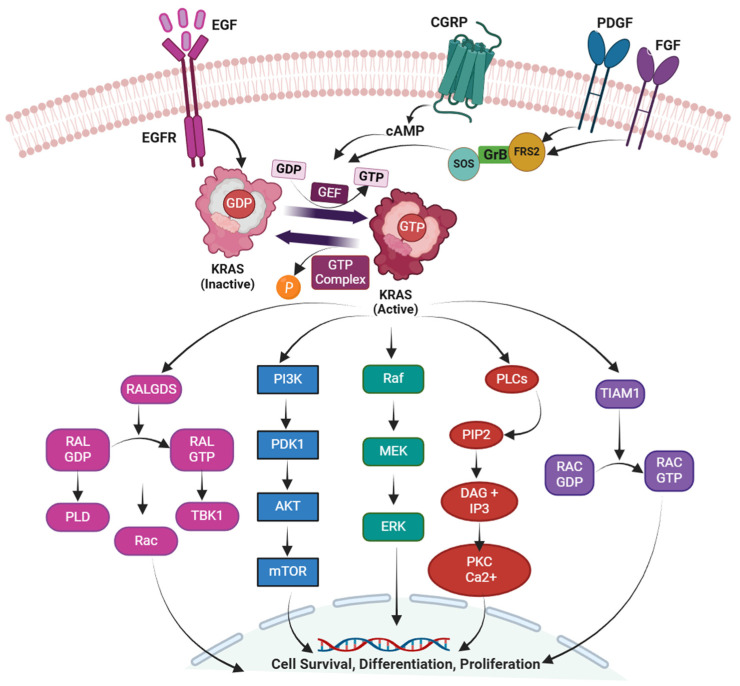
Analysis of KRAS downstream signaling pathways. In its inactive condition, KRAS associated with GDP. Ligands such as EGF, PDGF, and FGF initiate growth signals by activating receptor tyrosine kinases (e.g., EGFR), which recruit SOS via adaptor proteins (GrB and FRS2) to promote GDP-GTP exchange. Active KRAS (GTP-bound) triggers key signaling cascades, including RAF-MEK-ERK, PI3K-AKT-mTOR, RAL-GDS, and PLC pathways, facilitating cell survival, proliferation, and differentiation. EGF: epidermal growth factor; CGPR: calcitonin gene-related peptide; PDGF: platelet-derived growth factor; FGF: fibroblast growth factor; FRS2: fibroblast growth factor receptor substrate 2, GEF: guanine nucleotide exchange factors; RALGDS: ral guanine nucleotide dissociation stimulator; PLD: phospholipase D; TBK1: TANK-binding kinase 1; mTOR: mammalian target of rapamycin; RAF: rapidly accelerated fibrosarcoma; cAMP: cyclic adenosine monophosphate; GrB: granzyme B; SOS: son of sevenless; PI3K: phosphoinositide 3-kinases; MEK: mitogen-activated protein kinase; ERK: extracellular signal-regulated kinase; PLC: phospholipase C; PIP2: phospholipid phosphatidylinositol 4,5-bisphosphate; DAG: diacyl glycerol; IP3: inositol 1,4,5-trisphosphate; PKC: phosphokinase C; TIAM1: T cell lymphoma invasion and metastasis 1.

KRAS mutations may have multiple co-mutations, which can sometimes affect the functions of KRAS and the emergence and progression of tumors, along with the various mutation subtypes and mutation levels of various cancer tissues. Among 1078 NSCLC patients with KRAS mutations, 557 (53.5%) reported co-mutations. A study of 14 genes found that the tumor protein p53 (TP53) gene was the most often co-mutated, causing 39.4% of cases [80]. Specifically, 101 patients from the aforesaid cohort were evaluated for 14 supplementary genes, like the kelch-like ECH-associated protein 1 gene and the serine/threonine kinase 11 gene (STK11) [81]. Another study revealed that KRAS-mutated NSCLC shows EGFR mutations, although with a significantly lower frequency of less than 1% [82]. This is highly questionable due to the general belief that KRAS mutations and EGFR are independently exclusive [68,69]. More clinical findings will be required to develop more extensive and rational inferences in the future. Multiple subgroups had different physiological behaviors that influenced the health outcomes of patients [83]. For example, tumors containing a KRAS/STK11 co-mutation primarily possess a tumor microenvironment (TME) along with limited immune responses, missing the CD8+ tumor-infiltrating lymphocytes that comprise many T regulator cells. Activated dendritic cells and CD8+ tumor-infiltrating lymphocytes are widely present in TME with KRAS/P53 co-mutations, while T regulator cells are very few [84]. Consequently, the clinical evidence shows that patients with EGFR/KRAS or ALK commutations exhibit poor response to the treatment along with tyrosine kinase inhibitors [85]. About 16.6% of the patients in 11,951 Chinese tumor samples exhibited KRAS mutations, among which 14.5% possessed KRAS (G12C). The RAS/RTK pathway-related genomic irregularities were identified in approximately all patients (99.6%) with G12C mutations [86]. Additional gene abnormalities and individualized therapy are generally major considerations for treating KRAS-driven tumors (Figure 3).

### 3.2. Mutation Characteristics of Gene

**Figure 4:**
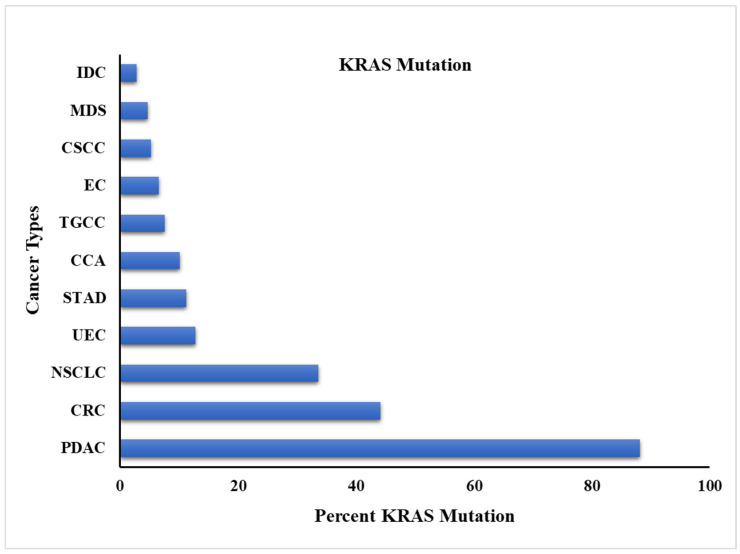
The KRAS mutation frequency in different cancers. PDAC: pancreatic cancer; NSCLC: non-small cell lung cancer; CRC: colorectal cancer; UEC: uterine endometrial cancer; STAD: stomach adenocarcinoma; CCA: cholangiocarcinoma; TGCC: testicular germ cell cancer; EC: esophageal cancer; CSCC: cervical squamous cell cancer; MDS: myelodysplastic syndrome, IDC: invasive ductal carcinoma.

KRAS is a part of the RAS family, which is regularly mutated, and it is suggested to be the oncogenic gene that prevalently drives human malignancies/cancers [3,54]. The most prominent KRAS mutations are observed in PDAC, NSCLC, and CRC [87]. The pattern of KRAS mutation varies considerably across various cancer types [9]. Most of the KRAS mutations included single-base missense mutations, with codon 12 (G12), codon 13 (G13), and codon 61 (Q61) contributing to 98% of all mutations [88]. Specifically, there are multiple malignancies in which KRAS mutations appear with multiple mutation frequencies. However, there is also a huge variety of mutant subtypes (Figure 4). For instance, KRAS mutations contribute about 67.6% of KRAS in PDAC, and the dominant mutant subtype is KRAS (G12D). Meanwhile, 20.6% of KRAS is in NSCLC, and the dominant substitution is G12C. It is essential to mention that the KRAS mutation rate in the pancreas was 67.6%, compared to the frequently mentioned 90%. Smokers between 25–35% probably have shown more KRAS mutations than non-smokers, who are reported only 5% [89]. Additionally, every KRAS mutation does not constitute driver mutations, and their patterns vary among non-smokers and smokers. For instance, the G12C KRAS mutation is commonly shown in regular-smoker patients, though KRAS (G12D) is commonly observed in the tumors of non-smoker individuals [90].

### 3.3. KRAS-Mutated Biochemical Heterogeneity

Modifications in the KRAS proteins are carried out via mutations to restrict KRAS from interacting with GAPs, including the hydrolyzing GTP linked with KRAS, keeping KRAS in a constantly activated state [58,59]. Several studies have demonstrated the physiological variation of KRAS mutation in various aspects, like the effector affinity, inherent GTPase activity, and metastatic regions. Hunter et al. reviewed that the modifications in the different codons 12, 13, and 61 mainly cause impairment of KRAS’s intrinsic GTPase activity, definitively with KRAS (G12C), KRAS (G13D), and KRAS (G12D) [91]. The correlation between KRAS and its effectors can also be affected by the kind of mutation [92]. Furthermore, KRAS (G13) is slightly delicate for neurofibromin 1, while KRAS (G12) and (Q61) are resistant/hypersensitive to the hydrolysis induced by neurofibromin 1, a kind of GAP [93]. Also, compared to cells with wild-type cells or genes with certain different KRAS mutations, cells consisting of KRAS (G12C) or (G12V) possess a higher extent of RAS-related protein A or B (RALA/B) signaling with a reduced extent of protein kinase B (AKT) phosphorylation [94]. Moreover, phosphorylated AKT is more prominent in the cell lines that contain KRAS (G12D) [95]. KRAS mutations can be classified into two groups depending on how efficiently they bind to RAF, a major effector, namely greater affinity (G12R, G12V, G12A, Q61L, and Q61H) and lower affinity (G12D, G12V, and G12R). The sensitivity of multiple KRAS-mutant subtypes towards targeted treatment differs. For instance, a finding using CRISPR-based engineering with higher fidelity discovered that KRAS (G12C) mutants primarily react to covalent-specific G12C inhibitors once EGFR is suppressed, whereas KRAS (G12D) mutants remain susceptible to EGFR inhibition in pancreatic human cancer model [96]. Regarding metastasis, patients having KRAS mutations are more susceptible to developing brain or lung metastases [97]. Following different experimental investigations, patients with G12V mutation generally possess pleuropericardial metastases, whereas individuals with G12C and G12D mutations primarily possess bone metastases. Generally, not all tumors with KRAS mutations are KRAS-dependent tumors. Extra applicable investigations are required to maximize the KRAS mutation’s heterogeneity map and establish a theoretical foundation for more effective individualized therapies.

These biochemical and physiological variations among KRAS mutations shape tumor growth and substantially alter the surrounding tumor microenvironment (TME), emphasizing the complicated interplay between genetic mutations and immune regulation.

## 4. Immune Escape Mechanism in Cancer Progression and Metastasis

### 4.1. KRAS Mutation and Tumor Microenvironment (TME)

Based on the various properties of KRAS mutations and the associated biochemical heterogeneity, it becomes evident that these genetic changes play a crucial role in altering the TME, enhancing immune evasion, and fostering tumor development and metastasis. There is significant evidence that genetic mutation patterns affect the immune environment of cancer. Increasing evidence in the literature reveals that KRAS mutation in tumors provides the intrinsic attributes of tumors, as well as contributes to the development of the tumor microenvironment (TME), which is significantly affected by the TME’s immune cells and ultimately contributes to immune escape and tumor progression [10,11].

### 4.2. KRAS-Driven Immune Modulation in Cancer

**Figure 5:**
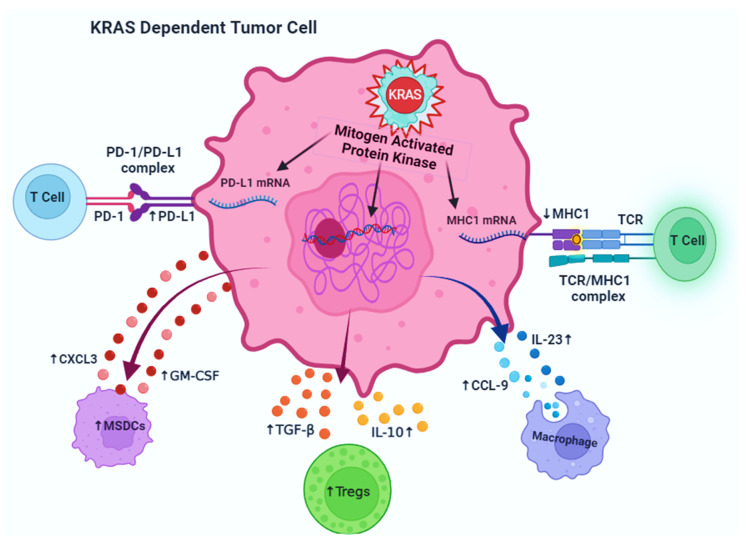
KRAS facilitates immune cell escape in the malignant macroenvironment by reducing MHC1 expression, increasing PD-L1 expression in cancer cells, and promoting the production of several types of chemokines and cytokines that target immunosuppressive immune cells. PD-1: programmed cell death 1; PD-L1: programmed cell death ligand 1; CXCL3: chemokine (C-X-C motif) ligand 3; MSDCs: myeloid-derived suppressor cells; GM-CSF: granulocyte macrophage colony-stimulating factor; TGF-β: transforming growth factor-β; IL-10: interleukin-10; CCL-9: C-C motif chemokine ligand-9; IL-23: interleukin-23; MHC1: major histocompatibility complex type 1; TCR: T-cell receptor.

Macrophages and T cells are two immune cells that are found to infiltrate the TME of a high proportion of solid tumors. Moreover, many of these immune cells, notably neutrophils, myeloid-derived suppressor cells (MDSCs), regulatory T cells (Tregs), tumor-associated macrophages (TAMs), and mast cells cultured/produced in TME, potentially exhibit immunosuppressive functions (Figure 5) [98]. The alteration of immune cells and the progression of an immunosuppressive TME also mainly rely on KRAS-mediated signaling. KRAS mutations modulate the intrinsic properties of the tumor cells, which regulate immune escape. Mutant KRAS regulates cancer immune escape in KRAS-driven tumors by enhancing PD-L1 expressions [99]. Emerging evidence suggests that lung cancers have high PD-L1 expression, including several KRAS (G12C), (G12D), (G12V), and (G13D) mutations [75,76]. Mutant KRAS signaling pathways improve the PD-L1 expression in tumor cells via the stability enhancement of PD-L1 mRNA [100,101]. However, AU-rich elements, mostly in PD-L1 mRNA 3’UTR, enable the AU-rich binding protein tristerrolin (TTP) to minimize PD-L1 production, causing the inhibition of TTP via downstreaming the MEK signal to the KRAS phosphorylates, enhancing PD-L1 expression levels [101]. A study revealed different methods for the KRAS (G12V)-mediated induction of PD-L1 expressions through stimulating ROS formation while promoting FGFR1 expressions [102]. The PD-L1 expressions of KRAS-mutant cells were substantially removed after antioxidant therapy, and FGFR1 gene downregulation diminished the cancer development and decreased PD-L1 expressions [102]. Unlike LAC, the intensity of PD-L1 expressions in patients suffering from CRC was not considerably affected by KRAS mutation. Additionally, influencing the PD-L1 expression, the KRAS (G13D) mutation in CRC cell lines reduced the communication of MHC class I molecules, which is detrimental to the immunogenicity of tumor cells [103]. MHC1 is required for antigen recognition and presentation through T cells. MHC 1 downregulation adversely diminished the ability of T cells, primarily CD8+ cytotoxic T cells, to remove tumors [104].

KRAS mutations modulate the immune cells in TME, especially the recruitment and acquisition of immune cell inhibitory phenotypes. KRAS mutation enhances the expression of identified cytokines, which convert into immunosuppressive Treg cells via producing CD4+ T cells in TME. It has been observed that the expression of IL-10 and TGF-β1 influenced through MEK/ERK/AP-1 signaling in CRC is a response of KRAS (G12C)-mediated phenotype conversion in T cells [105]. The gene excision of Treg cells in a lung cancer model of transgenic KRAS inhibited the formation and spread of lung tumors, showing the significance of Treg cells in the progression of lung cancers [106]. Adding to the point, the upregulation of GM-CSF in CRC and PDAC caused by KRAS (G12D and G12V) mutations elevated the MDSC’s infiltration of the TME and eventually resulted in immunological escape from antitumor immunity [107,108]. Findings from another research exhibited that KRAS (G12D) restricted interferon regulatory factor 2 (IRF2) secretion and enhanced CXCL3 production, sequentially acting on CXCR2 and MDSCs, as well as inducing the migration of MDSC to TME [109].

Along with KRAS mutation, alternative tumor suppressor genes or oncogenes in KRAS-mutated tumors may also be dysfunctional and involved in immune escape. The co-activation of KRAS (G12D) and MYC in lung cancer resulted in anti-inflammatory macrophage development, whereas T, B, and NK cells were not present because of the impacts of IL-23 and CCL9 [110]. Experiments on PDAC possess the stimulation of ARF6/AMAP1 pathways by TP53 and KRAS (G12D), affecting the magnitude and appearance of PD-L1 and also enhancing the tumor progression and immunological invasion [111]. Clinical data on LAC patients revealed that, when PD-1 inhibitors had been employed to diagnose people with KRAS, TP53, or KRAS/TP53-mutated cancer, the PD-L1 development in tumors, as in the TP53/KRAS co-mutation groups, was elevated, and the percentage of CD8+ T cells in the TME was significantly high [112]. Moreover, PD-1 inhibitory drugs were not successful in treating patients having STK11/LKB1 mutations in KRAS-mutant LAC, drawing attention to the novel pathway of resistance [102]. Thus, KRAS co-mutations frequently affect PD-L1 expression and the induction of immunosuppressive cells, significantly influencing how the immune system mediates the KRAS-driven TME.

KRAS mutations improve the immune-mediated landscape by influencing immune cell infiltration, increasing immunosuppressive properties, and encouraging immune checkpoint upregulation, notably PD-L1 expression. These alterations, together with the tumor mutation burden (TMB), tumor-infiltrating lymphocytes (TILs) inside the tumor microenvironment (TME), chemokines, and other oncogenic driver modifications, profoundly impact the clinical results of immune checkpoint blockade (ICB) therapy. By affecting the immune responsiveness of tumors, KRAS-driven functions emphasize the necessity of combining ICBs with complementary strategies, like chemotherapy, radiation therapy, or antiangiogenic agents, to improve the potency of immunotherapy and re-sensitize less-immunogenic tumors [113]. ARS-853, ARS-1620, MRTX1257, AMG-510 (sotorasib), and MRTX849 (adagrasib) are powerful KRASG12C inhibitors, with AMG-510 and MRTX849 being the initial compounds to enter clinical trials [114,115,116]. Whereas KRASG12C is widespread in lung cancer (13%), it is less prevalent in colorectal (3%) and other solid tumors (2%), underlining the necessity to target other KRAS mutations [117,118]. AMG-510 and MRTX1257 have produced a pro-inflammatory tumor microenvironment (TME), raised macrophage, dendritic cell, and T-cell density while encouraging CD8+ T-cell infiltration and tumor suppression. Combining AMG-510 with anti-PD-1 therapy has exhibited better survival in KRASG12C mutant models, [116,117] highlighting the role of TME regulation in KRAS-targeted treatments.

## 5. Immunotherapeutic Potential in KRAS-Mutant Tumors

Advanced cancer immunotherapy has made significant progress in recent years [119]. There have been reported significant clinical data that the survival rate of individuals with advanced tumors is substantially enhanced with immune checkpoint inhibitors (ICIs), specifically via inhibiting the immune checkpoint axis that participates in programmed death ligand 1 (PD-L1) and programmed cell death 1 (PD-1) [119,120]. The heterogeneous and complex TME is revealed to be associated with the responses to ICIs. [121,122]. The TME is classified into three categories depending on the degree of T-cell infiltration: the immune-inflamed phenotype, immune-excluded phenotype, and immune-desert phenotype [123,124]. After the administration of ICIs, an adequate anti-tumor response depends on the stimulation of clonal proliferation of antigen-exposed T cells in the TME. The infiltration and stimulation of effector T cells are inhibited by the presence of myeloid-derived suppressor cells (MDSCs), tumor-associated macrophages (TAMs), cancer-associated fibroblasts (CAFs), and regulatory T cells (Tregs) in the TME [125]. Therefore, treating ICIs in combination with other therapies that enhance the immunological effects of TME can be a preferable strategy for treating tumors. KRAS neoantigen-specific T-cell receptor (TCR)-adoptive T-cell therapy signifies a considerable advancement in the treatment of KRAS-mutated cancers. Recent investigations have found TCRs targeting the 9-mer KRAS-G12V mutant neoantigen in the context of human leukocyte antigen (HLA-A*11:01), exhibiting effective cytokine production, cytotoxic activity, and anti-tumor potency in preclinical settings. The unique structure of the KRAS-G12V peptide enhances its particular recognition by TCRs, indicating its application in tumor immunotherapy [126]. Furthermore, KRAS-G12V-specific TCR-engineered CD4+ T cells have shown excellent anti-tumor effectiveness in vitro and in xenograft models, identifying the neoantigens derived from HLA-DPB1*03:01 and DPB1*14:01. This kind of therapy has tremendous potential for targeted immunotherapy, particularly for solid malignancies [127,128].

Clinical studies of 4897 trials were carried out as of December 2021 to determine the PD-1/PD-L1 inhibitor efficacy. Approximately 83% of them utilize ICIs with some other treatments, including immuno-oncology treatments, radiation, and chemotherapy [129]. ICIs paired with chemotherapy had a superior therapeutic impact than ICIs alone, as per data from several clinical investigations [130,131]. Particularly in comparison to pembrolizumab monotherapy, the combination of a humanized monoclonal antibody pembrolizumab and platinum-based chemotherapy against PD-1 significantly enhanced the total survival of NSCLC patients, with PD-L1 ≥ 50% and negligible to gene mutations in the ALK and EGFR genes [132]. Recent clinical studies have indicated that TCR gene therapy addressing KRAS mutations, like KRAS-G12D, can reduce tumors. The patients with metastatic pancreatic cancer treated with modified T cells had a 72% partial response, giving significant support for the therapeutic potential of KRAS-targeted TCR treatments [133]. Recent developments in mRNA vaccine technology have increased their applicability beyond infectious diseases, including cancer immunotherapy. mRNA vaccines targeting neoantigens like KRAS G12V exhibit potential in advanced cancers by generating antigen-specific T-cell responses. Combining these vaccinations with pembrolizumab produced cancer reductions in advanced pancreatic and lung cancers, highlighting their therapeutic potential. [134]. ELI-002 2P, a cancer vaccine targeted against KRAS mutations, showed safe and effective T-cell responses in a phase 1 trial including 25 patients, indicating a reduction in tumor biomarkers and prolonged relapse-free survival in immunotherapy-resistant pancreatic and colorectal tumors [135].

Patients with KRAS-mutant PDAC, NSCLC, and CRC are presently being treated by mRNA-5671/V941, a vaccination targeted to the KRAS-mutant peptides (G12D, G12C, G13D, and G12V) (ClinicalTrials.gov identifier: NCT03948763). Upcoming outcomes will reveal essential information about the safety and efficacy of the mentioned combinations for KRAS-mutant tumors. A majority of therapeutic interventions that selectively target immune modulation in KRAS-mutant tumors have been examined in clinical trials, even though a broader range of investigative trials that impact various tumors have been examined, which are commonly directed through KRAS mutations, despite the mutation status (Table 2).

The clinical trials described emphasize current endeavors to advance immunotherapy for KRAS-mutated tumors across multiple carcinomas, including NSCLC, CRC, and PDAC. The specific mechanisms encompass PD-1/PD-L1, IL-1β, IL-6, JAK1/2, among others. Prominent drugs, including pembrolizumab, durvalumab, canakinumab, and spartalizumab, are undergoing evaluation either as monotherapies or in conjunction with medications such as EGFR inhibitors (e.g., selumetinib, trametinib), platinum-based chemotherapeutics, or novel modalities including mRNA vaccines (NCT03948763) and Coxsackievirus (CVA21, NCT02043665, and NCT02824965). Trials proceed from early-phase preliminary research (e.g., NCT03785249) to complex Phase III studies like CANOPY-1 (NCT03631199) and CANOPY-2 (NCT03626545). While therapies like pembrolizumab (NCT03991819, NCT01295827, and NCT02130466) have shown activity, the CRC shows decreasing susceptibility to monotherapies, owing to resistance mechanisms such as increased EGFR signaling (NCT03225664, NCT03299088). It emphasizes the need for combination methods. Additionally, sotorasib (NCT04185883) and adagrasib (NCT03785249), which are KRASG12C inhibitors, are being investigated in solid tumors, showing a potential to overcome KRAS-driven oncogenesis. Potential future options include targeting resistance pathways using dual or multi-targeted therapy, optimizing biomarkers like ctDNA for better patient inequality, and integrating innovative agents such as mRNA-based vaccines (NCT03948763). These trials attempt to refine individualized therapy options and improve survival results for patients with KRAS-mutated malignancies.

**Figure 6:**
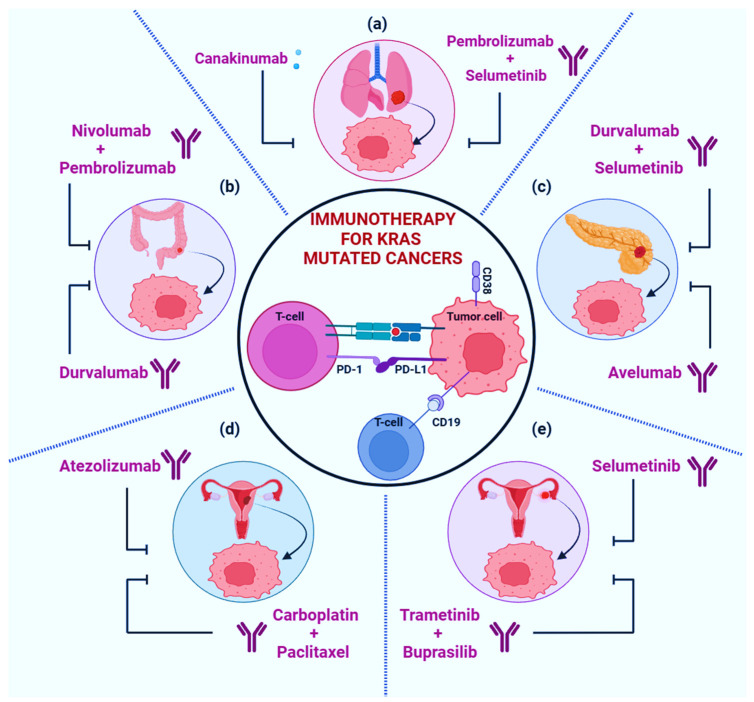
Immunotherapies targeting KRAS-mutated cancers are focused on immune-checkpoint markers such as programmed cell death 1 (PD-1), programmed cell death ligand 1 (PD-L1), and cytotoxic T-lymphocyte-associated protein 4 (CTLA-4). (**a**): Pembrolizumab + selumetinib (PD-1 inhibitors) and canakinumab (IL-1β inhibitor) in non-small cell lung cancers. (**b**): Nivolumab + pembrolizumab (PD-1 inhibitors) and durvalumab (PD-L1 inhibitor) in colorectal cancers. (**c**): Durvalumab + selumetinib (PD-1 and PD-L1 inhibitors) and Avelumab (PD-L1 inhibitor) in pancreatic cancer. (**d**): Carboplatin + paclitaxel (PD-1 and PD-L1 inhibitors) and atezolizumab (PD-L1 inhibitor) in endometrium cancers. (**e**): Trametinib + buprasilib (MEK1/2 and PI3K inhibitors) and celumetinib (MEK1 and MEK2 proteins inhibitor) in low-grade serous ovarian carcinoma.

In the domain of clinical cancer research, there is a significant focus on the immune microenvironment and its role in developing immunotherapeutic strategies that can stimulate an immune response against cancers [136]. At present, therapies that focus on immune-checkpoint markers, such as programmed cell death 1 (PD-1), programmed cell death ligand 1 (PD-L1), and cytotoxic T-lymphocyte-associated protein 4 (CTLA-4), are being employed in clinical research to deal with many types of cancers, including non-small cell lung cancer (NSCLC), colorectal cancer (CRC), pancreatic cancer, low-grade serous ovarian carcinoma (LGSOC), and endometrial cancer (EC) [137] (Figure 6).

### 5.1. Non-Small Cell Lung Cancer (NSCLC)

The predictive function of KRAS mutations in treated individuals for immune checkpoint inhibitors has been examined in various research. The function of KRAS throughout immunotherapy is not yet completely known. According to the meta-analysis by Kim et al., immunotherapy considerably increased overall survival compared to chemotherapy in pretreated KRAS-mutated NSCLC patients, except for individuals with wild-type KRAS [138]. However, the outcomes of contemplative research consisting of 530 formerly treated patients with NSCLC receiving nivolumab revealed that the KRAS status was not consistent evidence of immunotherapeutic efficiency in survival rates and response [139]. Chengming et al. revealed that KRAS-mutated NSCLC exhibited an inflammatory phenotype to adaptive immune tolerance, indicated via higher tumor mutational burden (TMB) and a higher percentage for CD8+ tumor-infiltrating lymphocytes (TILs) [140]. The experimental study of the KEYNOTE-042 trial in patients who were randomly preferred to receive pembrolizumab or first-line platinum-based chemotherapy revealed that the individuals who had KRAS G12C mutations had higher PD-L1 tumor proportion scores (TPS) and TMB in comparison to patients who had the KRAS wild-type mutation. Thus, the pembrolizumab-based approach may be recognized as a relevant comparator in clinical trials with first-line KRAS-targeted therapy on KRAS-mutated NSCLC depending on the efficacious outcomes in immunotherapy despite the KRAS mutational situation in this trial [141]. The associated appearance of certain other mutations, including a lack or modification of liver kinase B1 (LKB1)/serine–threonine kinase 11 (STK11), which serves as the driver gene of principle resistance to ICIs, can partially affect the putative immune sensitizing function of the KRAS mutation [142,143].

Furthermore, pembrolizumab, durvalumab, and atezolizumab have been accepted in clinics as first-line and second-line treatment therapies for patients having advanced NSCLC [120,144,145]. Moreover, as per clinical data on anti-PD treatment therapy, around 15–25% of NSCLC patients reported a response to ICIs [146]. The TME contains several components that provide drug resistance, and it has a significant impact on how the host responds to immunotherapy [147]. Targeted treatment therapy commonly possesses a substantial immunomodulatory effect on TME. Thus, targeted therapy is considered an effective combinational therapeutic strategy [148,149]. The novel KRAS (G12C) inhibitors enable the TME to shift from immunosuppressive to immunoreactive. Growing validations indicated that the KRAS mutations may mainly produce an immunosuppressive TME by facilitating the development of immunomodulatory markers in tumor cells, including interleukin-6, transforming growth factor, and interleukin-10 [150]. According to scientific research, treatment with AMG510 significantly enhances the dendritic cells, macrophages, and CD8+ T-cell infiltration in TME, notably CD103+ cross-presenting dendritic cells, which requires T-cell stimulation, preparation, and engagement [117]. The AMG510 treatment therapy generally elevates immunosurveillance and promotes the development of a proinflammatory microenvironment. Similarly, treatment with MRTX849 significantly enhanced immune-promoting M1-polarized macrophages, NKT and CD4 + cells, and dendritic cells. The decreased M2-polarized macrophages and intratumoral immunosuppressive myeloid-derived suppressor cells (MDSC) in KRAS (G12C) tumors, via modulating the tumor’s RNA expressions and protein, are involved in tumor antigen presentation or immunosuppressive TME mediation [151]. As per these observations, patients having KRAS mutations often possess an inflammatory TME and increased tumor immunogenicity, which causes greater response for PD-1/PD-L1 inhibitors [140]. Additionally, immune system stimulation is a prerequisite for the long-lasting antitumor response of KRAS (G12C) inhibitors. In immunocompetent animal models, treatment of large doses of AMG510 resulted in prolonged tumor regression, while tumors instantly recovered after the short response in immune-compromised mouse models [117]. The research above indicated that particular inhibitors targeting KRAS (G12C) in cancer cells fruitfully led to the transformation to an immunocompetent from an immunosuppressive TME. Combining immune checkpoint inhibitors with KRAS (G12C) inhibitors is a beneficial complementary approach to patients with KRAS (G12C)-mutated NSCLC.

Progress in treating KRAS-mutant NSCLC has some limitations that must be addressed. Immune checkpoint inhibitors (ICIs) demonstrate restricted efficacy, with 15–25% response rates, and their effectiveness varies among KRAS-mutant subtypes. Co-occurring mutations, such as LKB1/STK11, increase resistance, diminishing the immune-sensitizing capacity of KRAS mutations. The tumor microenvironment (TME) is a significant barrier, stimulating immunosuppressive variables such as MDSCs, M2-polarized macrophages, and cytokines, including IL-6, IL-10, and TGF-β, which restrict antitumor immunity. KRAS (G12C) inhibitors, such as AMG510 and MRTX849, have demonstrated potential by transforming the tumor microenvironment into an immunogenic state, improving immune cell infiltration and antigen delivery. However, combining KRAS inhibitors with immune checkpoint inhibitors is a synergistic strategy. However, it includes risks of increased toxicity and immunological-related side effects. Moreover, tumor heterogeneity and the fluctuating tumor mutational burden challenge patient diagnosis and long-term therapy results, highlighting the necessity for additional clinical research to enhance these techniques.

### 5.2. Colorectal Cancer (CRC)

Nowadays, immunotherapy, especially immune checkpoint inhibitors, has substantially improved cancer treatment [152]. Other specialized treatments suggest that immunotherapy may be more efficacious in some populations. In patients, microsatellite instability (MSI-high) and a dysfunctional DNA mismatch repair system (MMR) may have immunogenic potential. Researchers have reported sustainable responses to ICIs in patients having CRC [153].

The National Comprehensive Cancer Center Network (NCCN) has approved nivolumab and pembrolizumab for patient treatment with metastatic CRC in second-line and third-line settings based on the outcomes of two consecutive trials that showed the potential of PD-1 in blocking metastatic CRC [154]. In 2015, KEYNOTE-16418 highlighted the remarkable efficiency of pembrolizumab as a second-line or third-line therapy for MSI-H/MMR-deficient metastatic CRCs. A phase II clinical trial regulated by Le et al. studied the PD-1 inhibitor pembrolizumab (MK-3475) as a monotherapy on 41 previously treated patients having metastatic CRC, either with or without MMR deficiency. The 20-week progression-free survival (PFS) and immune-related objective response rate (ORR) were recognized as 78% and 40%, respectively, in MMR-deficient CRCs, compared to 0% and 11% in MMR-proficient CRCs. The median PFS and overall survival for patients with MMR-proficient CRC were 2.2 and 5.0 months, respectively, but not achieved in patients with MMR-deficient CRC. Patients with MMR-deficient non-CRC performed similarly to those with MMR-deficient CRC (71% ORR; 67% PFS). Prolonged PFS was associated with more significant somatic mutation burdens (*p* = 0.02) [155].

According to whole-exome sequencing, in MMR-deficient cancers, there were, on average, 1782 somatic mutations per tumor, in contrast to 73 in MMR-proficient tumors (*p* = 0.007). Nivolumab was utilized as a second and third-line treatment therapy in MMR-deficient/MSI-H metastatic CRCs in CHECKMATE-142 and was the third-largest trial to confirm the significance of immunotherapy in CRCs. During the 2016 ASCO Annual Conference, Overman et al. presented and described the preliminary findings of CHECKMATE-142 [156]. The outcomes were revised at the 2017 ASCO Gastrointestinal Cancers Conference [157]. None of the treatment-related fatalities occurred in this trial. These findings contribute to the FDA taking a further step and approving the first-ever insight into a biomarker instead of a specific cancer type. Pembrolizumab obtained rapid approval from the FDA for MMR-deficient or MSI-H cancer patients whose illness has developed after receiving standard therapy. The most prominent malignancies among individuals who had MSI-H were gastrointestinal, colorectal, and endometrial cancers. Breast, prostate, bladder, and thyroid malignancies were among the different tumors that have MSI-H and pembrolizumab activity [158]. AMG510 (sotorasib) and MRTX849 (adagrasib), inhibitors of KRASG12C, have demonstrated adequate safety and efficacy in preclinical and early-phase trials. However, their potency in colorectal cancer (CRC) is constrained by resistance mechanisms like enhanced EGFR signaling. Current combination therapy trials seek to improve outcomes in KRASG12C-mutated colorectal cancer (CRC) relative to non-small cell lung cancer (NSCLC) [159]. Many different clinical trial studies, mainly phase I and II trials, are ongoing with immunotherapy for metastatic CRCs (mCRCs) [153]. Hochster et al. determined the safety and effectiveness of bevacizumab (bev) and atezolizumab (atezo, PD-L1 inhibitor) in phase Ib trials with MSI-high metastatic CRC [160]. This combination had the expected toxicities and was well tolerated, whereas only a selected percentage of patients (between 5–15%) have mCRC, which is MSI-H/MMR-deficient. Immune checkpoint inhibitor therapy is believed to be the most beneficial for such individuals. The possible utilization of checkpoint inhibitors in larger patient groups with MSI-L or MSS is an area that is currently under investigation.

While immunotherapy offers potential for CRCs, certain limitations must be overcome for greater efficacy. A fundamental difficulty is the limited application of immune checkpoint inhibitors (ICIs) to microsatellite instability-high (MSI-H) or mismatch repair-deficient (MMR-deficient) CRCs, making up just 5–15% of metastatic CRC cases. Persons with microsatellite-stable (MSS) or mismatch repair-proficient (MMR-proficient) CRCs frequently show little response to ICIs, with much reduced progression-free survival and objective response rates. Resistance factors, such as increased EGFR signaling, further reduce the efficacy of KRASG12C inhibitors like AMG510 and MRTX849 in CRC relative to NSCLC. Combination therapy, including ICIs with bevacizumab or KRAS inhibitors, has proven efficacious in early trials but necessitates further validation to maximize safety and efficacy. Improving the potential use of ICIs for MSS CRC and addressing resistance mechanisms are significant areas for ongoing clinical trials.

### 5.3. Pancreatic Cancer

Pancreatic ductal adenocarcinoma (PDAC) possesses a small percentage of survival with few treatments available and a poor rate of therapeutic response [161]. This is primarily because pancreatic cancers have an immunosuppressive and fibrotic microenvironment [162]. KRAS mutations serve as the genes most associated with the alterations and genetic events that cause PDACs, appearing in over 90% of cancers [163]. KRAS G12D mutations in tumor cells have been proven to influence the pancreatic immune microenvironment via targeting regulatory T cells and immunosuppressive MDSCs [108,164,165,166]. A monotherapy of siltuximab was investigated as an anti-IL-6 monoclonal antibody in solid tumors with KRAS mutation but only produced minimal clinical benefits [167]. Siltuximab with the PD-1 inhibitor spartalizumab (PDR001) is a combination evaluated in phase I/II trials for metastatic pancreatic cancer (ClinicalTrials.gov identifier: NCT04191421). Upcoming outcomes will offer additional information regarding the efficacy of immunotherapy and anti-IL-6 therapy in combination. Patients who have KRAS-mutant solid tumors or metastatic PDAC are being treated with avelumab (PD-L1 inhibitor) and binimetinib (MEK inhibitor) in one clinical study (ClinicalTrials.gov identifier: NCT03637491). From the findings of the IMspire150 study, the US Food and Drug Administration (USFDA) finally sanctioned the administration of another PD-L1 inhibitor (atezolizumab) in combination with a BRAF inhibitor (vemurafenib) and a MEK inhibitor (cobimetinib) for the treatment of patients having advanced melanoma that has the BRAFV600 mutation (ClinicalTrials.gov identifier: NCT02908672). Depending on the research data, combining atezolizumab with vemurafenib and cobimetinib to targeted treatment therapy resulted in enhanced progression-free survival and a decreased related risk for development or death [168]. These outcomes suggest a possible synergistic relationship between ICIs, BRAF, and MEK inhibitors. In recent years, the new compound MRTX849, created by Mirati Therapeutics, has commenced clinical trials as a selective covalent inhibitor of KRASG12C [169].

Notably, therapeutic strategies combining radiation and/or chemotherapy with immune checkpoint inhibitors demonstrated promising outcomes (Table 2). Highly humanized anti-CTLA-4 IgG1 monoclonal antibody (mAb) ipilimumab was sanctioned for clinical usage in Europe and the USA in 2011 [170]. Consequently, the outcomes in patients having locally developed or metastatic PDAC have been unsatisfactory [171]. Furthermore, ipilimumab treatment in PDAC patients has been connected with enhanced toxicity [171]. Surprisingly, chemotherapy in combination has produced additional promising outcomes. In addition, by promoting naive T-cell activation, a standard therapy of gemcitabine for advanced PDAC has shown excellent immunological response [172,173]. The preliminary conclusion from the phase Ib clinical trial (NCT01473940) combining ipilimumab and gemcitabine indicated a PFS of 2.5 months (95% CI 0.8–4.8) and a median overall survival of 8.5 months (95% CI 2.2–10.3) [174,175]. A human IgG2 mAb named tremelimumab (anti-CTLA-4 inhibitor) was examined as a monotherapy in the phase II open-label investigation (NCT02527434) [176]. Tremelimumab produced unsatisfactory results, with an average overall survival of 4 months (95% CI 2.83–5.42) in 18 of 20 patients having progressive disease. Moreover, Aglietta et al. investigated the efficacy of tremelimumab in combination with gemcitabine in a phase I study (NCT00556023) [177]. In 28 out of 34 patients, the tumor response was determined, and the median overall survival time was 7.4 months (95% CI: 5.8–9.4). Seven individuals exhibited SD for more than 10 weeks with acceptable toxicity, while two individuals attained PR [177]. In a phase II (part A) clinical investigation of tremelimumab, in combination with the anti-PD-L1 drug durvalimab, was investigated as second-line therapy followed by the failure of gemcitabine-based chemotherapy. Thus, according to an efficacy assessment in comparison to durvalumab monotherapy, the combination possesses an objective response rate (ORR) of 3.1% (95% CI 0.08–16.22) [178]. The required threshold efficacy (10%) was not obtained in Part A. Consequently, Part B of the research, which was organized to be a randomized or non-randomized clinical trial based on the findings of Part A, was not performed. According to immunohistochemical research, pembrolizumab’s PD-L1 expression is associated with lower OS in PDAC [179]. The immunomodulatory effects of gemcitabine and paclitaxel, a popular first-line chemotherapy combination for metastatic PDAC, have been documented earlier [180]. Pembrolizumab, a PD-1 inhibitor, was investigated in a phase Ib/II trial (NCT02331251) to examine its safety and effectiveness when combined with nab-paclitaxel chemotherapy and gemcitabine [181]. Overall survival and PFS were measured at 15.0 and 9.1 months, respectively, which demonstrated a significant relationship between overall survival and PFS (r = 0.777), whereas only 53% of patients received grade-3 events [181].

Pancreatic cancer represents notable limitations in treatment because of its highly immunosuppressive and fibrotic microenvironment. Clinical trials integrating immunotherapy with chemotherapy, like ipilimumab with gemcitabine or tremelimumab with gemcitabine, have demonstrated minimal survival benefits and severe toxicity. Combining immune checkpoint inhibitors (ICIs) with targeted therapy, like MEK and BRAF inhibitors, results in positive results in other cancers, but its inclusion in PDAC has been less favorable. The immunological responses are still moderate, even though combination treatments, such as anti-IL-6 monoclonal antibodies with PD-1 inhibitors, are still being studied. The lack of efficient biomarker-driven patient selection and the ability of cancers to overcome immune responses provide poor findings in PDAC immunotherapy trials.

### 5.4. Low-Grade Serous Ovarian Carcinoma (LGSOC)

LGSOC constitutes 5–10% of all serious ovarian carcinomas and particularly unique kinds of epithelial primary or ovarian primary peritoneal tumors [182]. However, LGSOC has a more flexible biological function relative to its high-grade counterpart. Nevertheless, it frequently reflects chemotherapy resistance [182]. The most frequent somatic changes (~40%) are the activated mutation in the MAPK pathways, with KRAS mutations appearing in around 15–54% of LGSOCs throughout several case groups [183]. RAS/RAF/MEK pathways inhibitors have been examined for the patients with LGSOC [184]. A phase II study comprising 52 patients having LGSOC (14 patients had KRAS mutant and 2 patients had BRAF mutant) showed that selumetinib had an 80% DCR, a median PFS of 11 months, and a 2-year overall survival of 55% [185]. In a phase Ib trial evaluating the combination of trametinib (a MEK1/2 inhibitor) and buparlisib (a PI3K inhibitor), patients with LGSOC produced a DCR of 76.2% and a response rate of 28.6% [186].

A limitation in the treatment of low-grade serous ovarian carcinoma (LGSOC) includes recurrent chemotherapy resistance, despite the tumor’s variable cellular activity. While inhibitors addressing MAPK and RAS/RAF/MEK pathways exhibit some efficacy, the mechanisms behind drug resistance are not entirely understood. Additionally, the variety of somatic mutations in LGSOC hampers the discovery of an effective, uniform therapy.

### 5.5. Endometrial Cancer (EC)

In developed nations, EC is the most prevalent malignancy in the genital tract of females [187]. The two kinds of ECs. Type I ECs are typically small-to-intermediate-grade tumors that usually exhibit estrogen receptors, whereas type II ECs appear as a high-grade illness having more early stages at presentation [188]. The KRAS mutation is usually linked to microsatellite-instability (MSI) EC and has been linked to type I EC (10–30%) [189,190]: The Tumor Cancer Genome Atlas (TCGA) investigation of EC patients reported that KRAS-mutated ECs have a greater and more significant estrogen signaling activation due to enhanced RAS/MAPK pathway signaling. This condition may have therapeutic implications since it shows that anti-estrogen medication may be necessary to treat KRAS-mutant EC as effectively as possible [191]. Selumetinib failed to meet the pre-trial requirements for clinical effectiveness in the phase II study in women having recurrent EC [192]. An analysis of the pathways involved in disease shows that single-agent targeted treatment may not provide significant clinical improvements. Although there are limited clinical findings, several combination approaches have been explored, including combining Akt inhibitors with MEK inhibitors and Akt or PI3K/mTOR inhibitors with poly-ADP ribose polymerase (PARP).

Challenges to the treatment of endometrial cancer (EC) are various regarding the tumor’s biology, particularly between type I and type II ECs, which influences treatment options. Considering the link between KRAS mutations and microsatellite instability in type I EC, the success of targeted medicines like selumetinib has been limited with phase II studies not reaching their therapeutic objectives. Furthermore, the intricacy of the RAS/MAPK and estrogen signaling pathways suggests that single-agent treatments may not generate meaningful clinical improvements, which requires additional research on combination therapy.

## 6. Conclusions

Exploring the complex domain of addressing KRAS signaling pathways and their effects in chemotherapy reveals a wide range of opportunities in various kinds of cancers. KRAS-mutant pancreatic cancer, KRAS-driven lung carcinomas, and KRAS-altered colorectal cancers all provide distinct problems and chances for intervention due to their invasive nature, resilience, and complexity. Immuno-therapeutic strategies are particularly promising in this context. Utilizing the immune system to target cancer cells with KRAS mutations specifically has the potential to overcome resistance mechanisms and improve the effectiveness of treatment. By integrating immunotherapies with targeted medicines designed to interfere with KRAS signaling pathways, it is possible to achieve synergistic effects that enhance patient outcomes and increase survival rates. However, being in the early stages, current research efforts investigating these immune-based treatment approaches in KRAS-mutant tumors are expected to provide extremely useful knowledge, influencing the development of personalized cancer treatments in the future. The combination of immunotherapy and targeted therapy against KRAS represents a significant advancement in precision medicine, providing optimism for improved therapies for patients with various types of KRAS-mutant malignancies.

## 7. Future Perspectives

Due to the prominent occurrence of the KRAS mutation and its function in inducing and promoting tumor formation, targeting KRAS is a promising approach. It was initially believed to be impossible to target KRAS directly due to its specific properties. The improvement of KRAS-targeting therapies has been supported by various important insights, achieved via active exploration, that have advanced our awareness of KRAS mutations.

The current article describes an insight that goes beyond the oncogenic KRAS signals that promote development and explains various ranges of validations, supporting the idea that it modulates the immune microenvironment in several different mechanisms. Certain oncogenic KRAS mutation-induced alterations in cancer enhance tumor cell proliferation (mitogenic effects). In contrast, other immunomodulatory alterations facilitate tumors escaping immune-mediated invasion via developing an immunosuppressive microenvironment.

However, it gives a perspicuous idea about how KRAS-induced immunomodulation impacts tumorigenesis, which has commonly been enhanced during the past years. Further research is still required to understand how oncogenic KRAS-containing cells interact with their microenvironment fully. In previous years, the primary focus of research was on the KRAS-mediated events targeting lung and pancreatic tumors. It is also suggested that exploring these pathways in different cancer types, where KRAS mutations are less prevalent, is necessary. Improved knowledge of KRAS-mutated outcomes and their targeted therapeutics could support minimizing resistance to immunotherapy. Consecutively, it is preferred to determine and differentiate the patients who might gain an advantage from immunotherapies and confirm the eventual achievement of KRAS-targeted medicines, and it will be essential in future research to reveal tumor-specific KRAS-induced pathways. Additionally, identifying the KRAS downstream effector mechanism will be important in the new efficacious combination approach.

## Figures and Tables

**Table 1:** PDB IDs of submitted KRAS protein structures in protein databank with bound ligands and possible mutations.

S. No.	Nucleotide Ligand/Cofactor Bound to KRAS Protein	Mutation in Protein Structure	PDB IDs
1	GDP	WT	4LPK
2	G[D/C]P	WT	5UK9
3	GNP	WT	5UFE
4	GDP	WT + E	5W22
5	GSP	WT + E	5VQ6
6	GTP	WT + E	5VQ2
7	GDP	G12A + E	5VP7
8	GNP	G12A + E	5VPY
9	GSP	G12A + E	5VPZ
10	GTP	G12A + E	5VPI
11	GDP	G12C	5V71
12	N/A	G12C	5KYK
13	GDP	G12C + E	4M22
14	N/A	G12A + E	6EPM
15	GDP	G12D	5US4
16	GCP	G12D	6QUV
17	GDP	G12D + E	4EPR
18	GCP	G12D + E	6GJ5
19	GDP	G12R	4QI3
20	GNP	G12R	6CU6
21	GDP	G12V	5VQW
22	GNP	G12V	6GOE
23	N/A	G12V	6H47
24	GDP	G12V + E	4PZZ
25	GNP	G12V + E	5WHE
26	GDP	G13D	4TQA
27	GNP	G13D	6E6F
28	GCP	A59G	6ASE
29	GDP	E	5VBM
30	GDP	Q61A + E	5VQ1
31	GNP	Q61H	6F76
32	GNP	Q61H + E	5OCT
33	GDP	Q61L	4WA7
34	GDP	A146T	6BOF
35	N/A	HVR	1KZP
36	(a) ASP6918—IUPAC Name: (1-[7-[6-ethenyl-8-ethoxy-7-(5-methyl-1~{H}-indazol-4-yl)-2-(1methyl piperidine-4-yl)oxy-quinazolin-4-yl]-2,7-diazaspiro [3.5]nonan-2-yl]propan-1-one) (b) GDP	G12C	8 × 6R
37	(a) BI-5747—IUPAC Name: ((3~{S})-5-oxidanyl-3-[2-[[6-[[3-[(1~{S})-6-oxidanyl-3-oxidanylidene-1,2-dihydroisoindol-1-yl]-1~{H}-indol-2-yl]methylamino]hexylamino]methyl]-1~{H}-indol-3-yl]-2,3-dihydroisoindol-1-one) (b) GCP	G12D	7ACH
38	(a) BI-0474—IUPAC Name: (2-amino-4,5,6,7-tetrahydro-1-benzothiophene-3-carbonitrile) (b) GDP	G12C	7U8H
39	(a) MRTX0902—IUPAC Name: (2-methyl-3-[(1R)-1-{[4-methyl-7-(morpholin-4-yl)pyrido [3,4-d]pyridazin-1-yl]amino}ethyl]benzonitrile)	SOS1:KRAS G12C	7UKR
40	(a) AMG 510—IUPAC Name: (6-fluoro-7-(2-fluoro-6-hydroxyphenyl)-1-(4-methyl-2-propan-2-ylpyridin-3-yl)-4-[(2S)-2-methyl-4-prop-2-enoylpiperazin-1-yl]pyrido[2,3-d]pyrimidin-2-one) (b) GDP	G12C	6OIM

**Table 2:** Clinical trial studies of immunotherapy in various KRAS-mutated tumors (assessed in August 2024).

Cancers	Mechanism /Target(s)	Drug Compound (s)	Combination Drug Compound (s)	Trials	Trial Identification Number	Current Status
NSCLC	PD-1	SHR-1210	Apatinib	II	NCT0377712 4	Not yet recruiting
NSCLC	PD-1	Pembrolizumab	Selumetinib	I	NCT0329908 8	Recruiting
NSCLC	PD-1	Pembrolizumab	Trametinib	I/II	NCT0322566 4	Recruiting
NSCLC	PD-1	Pembrolizumab	Binimetinib	I	NCT0399181 9	Active, not recruiting
NSCLC	PD-1	Durvalumab (MEDI4736)	Selumetinib	II	NCT0300410 5	Withdrawn (insufficient funding)
NSCLC	IL-1β	Canakinumab	—	III	NCT0344776 9—(CANOPY-A)	Recruiting
NSCLC	IL-1β or/and PD-1	Canakinumab or/and pembrolizumab	—	II	NCT0396841 9—(CANOPY-N)	Recruiting
Metastatic NSCLC	JAK1/2	Momelotinib	Trametinib	I	NCT0225860 7	Terminated
Metastatic pancreatic cancer	IL-6 and PD-1	Siltuximab (CNTO 328) and Spartalizumab (PDR001)	—	I/II	NCT0419142 1	Recruiting
CRC, TNBC, NSCLC	PD-1 and IL-17 or IL- 1β	Spartalizumab (PDR001) and CJM112 or canakinumab	Trametinib	I	NCT0290066 4	Active, not recruiting
NSCLC and bladder cancer	—	CVA21 (Coxsackievirus) and Pembrolizumab	—	I	NCT0204366 5	Completed
NSCLC and HCC	IL-8 and PD-1	Anti-IL-8	Nivolumab	II	NCT0412337 9	Recruiting
Metastatic or unresectable locally advanced melanoma	—	Atezolizumab	Cobimetinib, vemurafenib	III	NCT0290867 2 (IMspire150)	Active, not recruiting
Metastatic carcinoma, melanoma, or NSCLC	PD-1	Pembrolizumab	—	I	NCT0129582 7 (KEYNOTE-001)	Completed
Metastatic cancer	IL-1R	Anakinra	—	I	NCT0007211 1	Completed
Advanced melanoma and solid tumors	PD-1	Pembrolizumab	Trametinib, dabrafenib	I/II	NCT0213046 6	Active, not recruiting
Advanced malignant solid tumors	IL-8	BMS-986253 (HuMax-IL-8)	—	I	NCT0253646 9	Completed
Solid tumors	PD-1	Pembrolizumab	MRTX849	I/II	NCT0378524 9	Recruiting
Solid tumors	IL-6	Siltuximab (CNTO 328)	—	I/II	NCT0084119 1	Completed
Solid tumors	PD-1 or PD- L1	PD-L1 or PD-1 inhibitors	Sotorasib (AMG 510)	I	NCT0418588 3	Recruiting
Metastatic CRC	—	Durvalumab	—	II	NCT0222766 7	Recruiting
Metastatic CRC	—	Pembrolizumab	mFOLFOX6	II	NCT0237567 2	Ongoing; not recruiting
Metastatic CRC	—	Pembrolizumab	Radiotherapy/ablation	II	NCT0243707 1	Recruiting
Chemorefractry Metastatic CRC	—	Pembrolizumab	Azacitidine	I	NCT0226044 0	Ongoing; not recruiting
Metastatic CRC	—	Pembrolizumab	—	II	NCT0187651 1	Recruiting
NSCLC	IL-1β or/and PD-1	Pembrolizumab or/and Canakinumab	Platinum-based doublet chemotherapy	III	NCT0363119 9—(CANOPY-1)	Not recruiting
NSCLC	IL-1β	Canakinumab	Docetaxel	III	NCT0362654 5—(CANOPY-2)	Active, not recruiting
NSCLC	IL-1β and PD-1	Spartalizumab (PDR001) and canakinumab	Platinum-doublet chemotherapy	I	NCT0306485 4	Not recruiting
NSCLC	—	CVA21 (Coxsackievirus) and pembrolizumab	—	I/II	NCT0282496 5	Active, not recruiting
PDAC	—	Momelotinib	Capecitabine and oxaliplatin	I	NCT0224448 9	Terminated
NSCLC, CRC, PDAC	PD-1	Pembrolizumab	mRNA-5671/V941	I	NCT0394876 3	Recruiting
Metastatic PDAC and KRAS- mutant solid tumors	PD-L1	Avelumab	Binimetinib	I/II	NCT0363749 1	Recruiting

## List of Abbreviations

KRAS	Kirsten rat sarcoma viral oncogene homolog
TME	Tumor microenvironment
NRAS	Neuroblastoma rat sarcoma virus
HRAS	Harvey rat sarcoma virus
MAPK	Mitogen-activated protein kinase
PI3K	Phosphoinositide 3-kinase
RAL-GEF	Ral guanine nucleotide exchange factors
NSCLC	Non-small cell lung cancer
PDAC	Pancreatic ductal adenocarcinoma
CRC	Colorectal cancer
LAC	Lung adenocarcinoma
EGFR	Estimated glomerular filtration rate
GRB2	Growth factor receptor-bound protein 2
GEF	Guanine nucleotide exchange factor
MAP/ERK	Mitogen-activated protein kinases/extracellular signal-regulated kinases
NMDAR	N-methyl-D-aspartate receptor
Camp	Cyclic adenosine monophosphate
ShP2	Src homology region 2 (SH2)-containing protein tyrosine phosphatase 2
PDK1	3-Phosphoinositide-dependent kinase 1
MDSCs	Myeloid-derived suppressor cells
TMB	Tumor mutational burden
TILs	Tumor-infiltrating lymphocytes
PD-1/PD-L1	Programmed cell death Ligand 1
